# 甘氨酸诱导细菌外膜囊泡分泌的脂质组学分析

**DOI:** 10.3724/SP.J.1123.2024.10017

**Published:** 2025-05-08

**Authors:** Jingyuan SONG, Xiulei QI, Huaizhong GUO, Lianghai HU

**Affiliations:** 1.河北大学药学院, 河北 保定 071002; 1. College of Pharmaceutical Sciences, Hebei University, Baoding 071002, China; 2.吉林大学生命科学学院, 吉林 长春 130023; 2. School of Life Sciences, Jilin University, Changchun 130023, China

**Keywords:** 细菌外膜囊泡, 离子淌度质谱, 脂质组学, 甘氨酸, bacterial outer membrane vesicles, ion mobility mass spectrometry, lipidomics, glycine

## Abstract

细菌外膜囊泡(outer membrane vesicle, OMV)是由细菌分泌的具有双层磷脂膜结构的外囊泡,携带多种亲本细菌生物活性物质,可作为疾病标志物,因其具有良好的生物相容性,在作为抗癌抗菌药物载体方面也具有很大的潜力。在大肠杆菌培养过程中添加甘氨酸可促进细菌OMV的分泌,但诱导所引起细菌OMV的脂质成分差异未见报道。本研究对甘氨酸诱导前后的细菌OMV的关键质膜组成部分进行脂质组学分析,利用两亲性树枝状聚合物超分子探针对细菌培养上清液进行细菌OMV的捕获,在细菌OMV数量保持一致的条件下,采用甲基叔丁基醚脂质提取法对甘氨酸诱导前后的细菌OMV进行脂质提取。采用超高效液相色谱-离子淌度-飞行时间高分辨质谱仪(UPLC-IMS-QTOF-MS)和MS-DIAL(Mass Spectrometry Data Independent Analysis for Lipidomics)脂质数据分析软件对甘氨酸诱导前后的细菌OMV脂质组成进行分析,确定甘氨酸诱导后细菌OMV关键质膜上的差异脂质种类。研究发现,在大肠杆菌培养过程中添加甘氨酸会引起其大肠杆菌OMV表面和内部脂质成分发生显著变化,神经酰胺(Cer)和溶血磷脂酰胆碱(LPC)的表达呈现出显著升高,双(单酰基甘油)磷酸酯(BMP)的表达显著降低,而甘油三酯(TG)和鞘磷脂(SM)的表达呈现无规律的变化,值得下一步深入探索其调控机制。本研究可为后续的OMV脂质成分深入研究和用于新型药物递送载体的研究提供一定参考。

## Abstract (en)

Outer membrane vesicles (OMVs) are nanoparticles with double-phospholipid membrane structures that are secreted by gram-negative bacteria and carry a variety of bioactive substances from parental bacterial cells; consequently, OMVs serve as disease markers. Moreover, bacterial OMVs are potential anticancer- and antibacterial-drug carriers. While the addition of glycine during bacterial culturing promotes the secretion of bacterial OMVs, glycine-induced differences in the lipid compositions of such OMVs have not yet been reported.

In this study, the key plasma membrane components of bacterial OMVs before and after glycine induction were analyzed using lipidomics. Bacterial OMVs were captured from bacterial-culture supernatants using an amphiphilic dendritic polymeric supramolecular probe. Two sets of enriched bacterial OMVs were characterized and their enrichment efficiencies determined, after which the numbers and purities of the OMVs within the samples were determined using a nanofluidic assay. Lipids were extracted using the methyl *tert*-butyl ether lipid-extraction method when consistent numbers were recorded. The lipid compositions of the bacterial OMVs before and after glycine induction were analyzed using an ultra-performance liquid chromatography-ion mobility spectrometry-quadrupole time-of-flight mass spectrometer (UPLC-IMS-QTOF-MS) and MS-DIAL software. Differential lipid species in the key plasma membranes of the bacterial OMVs following glycine induction were recorded along with their corresponding amounts. Detection was accomplished in positive-ion scanning mode using an ACQUITY UPLC BEH C18 column following UPLC-MS injection, and MS^E^ mass-spectrometry data-acquisition mode. The lipid components in the two groups were determined by combining mass-spectrometric and software-analysis data, which revealed that the addition of glycine to the *E. coli* Nissle 1917 culture led to two-to-three-times higher concentrations of OMVs than observed for the untreated group under the same culturing and enrichment conditions. Particle numbers measured for the same volume revealed one-order-of-magnitude more bacterial OMVs after induction than before, with the treated group exhibiting slightly larger particles (on average); however, these particles were better dispersed and less likely to aggregate. The identified lipid components were categorized to determine the amount of each lipid type. Differentially expressed lipids were subsequently screened according to experimental conditions; significantly different expression levels were observed following glycine induction, with 820 lipids identified among the 10165 components detected. The lipid classes were ranked in order of quantity as: glycerolipids (GL), fatty acids (FA), sphingolipids (SP), glycerophospholipids (GP), saccharolipids (SL), and sterol lipids (ST), among which 463 GL lipid fractions (56.4% of all characterized lipids) were recorded. The ST lipid fraction contained the fewest members (10) and qualitative lipids were determined to make up 1.2% of the total. The addition of glycine to the *E. coli* culture was found to induce significant changes in the surface and internal lipid composition of the *E. coli* OMVs, with significantly more ceramide (Cer) and lysophosphatidyl choline (LPC), and significantly less bis(monoacylglycerol)phosphate (BMP) expressed. Partial triglyceride (TG) and sphingomyelin (SM) were irregularly expressed following glycine treatment, with equal amounts of up- and down-regulated lipids observed. This study provides a reference for subsequent in-depth studies into the lipid compositions of OMVs and their use as novel drug-delivery carriers. The expression of TGs and SM showed irregular changes, which is worthy of the next step of in-depth exploration of its regulatory mechanism, and the present study provides a certain reference for the subsequent in-depth study of the lipid composition of OMV and its use in the study of novel drug delivery carriers.

细菌外膜囊泡(outer membrane vesicle, OMV)是由革兰氏阴性菌分泌的具有双层膜结构的纳米级囊泡^[1]^,在细菌生长的各个阶段均可分泌。OMV通过细菌群体感应在细胞通讯中发挥重要作用^[2]^,并携带多种亲本细菌细胞生物活性物质^[3]^,可反映其母体细胞的生理和病理状态。因其具有高生物相容性、长循环时间和包封生物分子的固有能力^[4]^,在作为免疫刺激剂、疫苗和抗癌、抗菌药物递送载体^[5,6]^方面具有很大的潜力和吸引力。目前OMV主要来源于细菌培养液,因培养条件简单,周期短,适合进行规模化生产。*Escherichia coli* Nissle1917(EcN)是革兰氏阴性益生菌株^[7]^(为方便阅读,下文均以Nissle作为通用缩写),可定植并保护肠道,在医学上可用于治疗感染性和炎症性肠道疾病^[8,9]^,目前已有相关药品上市^[10]^。主要原理是EcN细菌的H1型鞭毛通过细菌之间的相互作用以及锚定识别作用,在肠道上皮表面形成一层紧密的网络结构,从而阻止病原菌对肠道上皮细胞的黏附及侵袭^[11]^。此外其表面的脂蛋白还可增强宿主细胞介导的免疫应答^[12]^,调节促炎性和抗炎性细胞因子之间的平衡。

为实现OMV的高纯度和高回收率分离,多种分离方法应运产生,常见的纯化富集手段有超速离心^[13]^、密度梯度离心^[14]^、尺寸排阻色谱^[15]^和分子印迹法^[16]^等。其中,超速离心法被认为是OMV分离手段的金标准。尽管这些方法可基本满足OMV的富集,但操作复杂,耗时较长。为实现OMV简便快速的分离,近年来,研究者开发了高通量的OMV富集方法,如Feng等^[17]^制备了一种两亲性树枝状聚合物超分子探针(amphiphile-dendrimer supramolecular probe, ADSP),可有效插入OMV的磷脂双分子层膜,进而实现对OMV的高效捕获与分离,并可进行原位的蛋白质免疫检测。Shi等^[18]^发展了一种从大肠杆菌磁性收获OMV的高产率方法,大肠杆菌在聚乙二醇(PEG)化磁性氧化铁纳米颗粒(MNPs)存在下生长,可增加细菌的MNPs摄取和磁性OMV分泌,从而实现高产量磁性收获OMV。OMV由脂质、蛋白质、核酸和小分子代谢物质组成,用于细胞间生物信息的传递^[19]^,在一定程度上也可反映机体生理状态的变化。甘氨酸,又名氨基乙酸,在细菌培养过程中加入可促进细菌外排泵工作,进而表现为OMV分泌的增加,但此过程也伴随着细菌内部物质共流出^[20]^。研究发现,在细菌培养液中添加质量分数为1%的甘氨酸,可显著促进OMV的分泌,对甘氨酸诱导前后分泌的OMV进行形态学和免疫学表征,结果表明两者均可引起强烈的免疫反应,但诱导后所分泌OMV的表面毒素活性显著降低^[21]^。虽然甘氨酸可诱导细菌分泌更多的OMV,但和正常条件下分泌的OMV的成分差异未见报道。OMV的双层膜由多种脂质构成,对其脂质组成的分析可直接反应甘氨酸诱导前后OMV组成的变化,为OMV用于药物递送的研究提供重要参考。同时脂质代谢与机体疾病密切相关,且多数脂质代谢物具有良好的稳定性,因此它也常作为疾病生物标志物^[22]^。本研究拟采用超高效液相色谱-高分辨质谱(UPLC-HRMS)对OMV进行非靶向脂质组学分析,通过比较甘氨酸诱导前后OMV的脂质组差异,考察甘氨酸诱导前后OMV的脂质变化规律,为后续OMV脂质成分的深入研究和作为药物递送载体的应用提供一定参考。

## 1 实验部分

### 1.1 仪器、试剂与材料

ACQUITY UPLC H-Class PLUS液相色谱仪-SYNAPT XS质谱仪(UPLC-IMS-QTOF-MS,美国Waters); CentriVap真空离心浓缩仪(美国Labconco); Zetasizer Pro纳米粒度和ZETA电位分析仪(英国Malvern Panalytical);纳米流式检测仪(英国APPOGE);全波长光吸收酶标仪(奥地利Tecan Austria Gmbh)。

氨水(28%)购自国药集团化学试剂有限公司(上海); 1-乙基-(3-二甲基氨基丙基)-3-乙基碳二亚胺(EDC)、*N*-羟基琥珀酰亚胺(NHS)、脂肪酸甲酯磺酸钠(MES)、乙腈(ACN,色谱级)、甲醇(色谱级)、异丙醇(IPA,色谱级)、二甲基亚砜(DMSO, >99%)、甲基叔丁基醚(MTBE,色谱级)、脂质标准品鞘磷脂(SM, >99%)购自阿拉丁生化科技股份有限公司(上海);磷酸缓冲液(PBS)购自普诺赛生命科技有限公司(武汉); BCA蛋白质定量试剂盒购自碧云天生物技术有限公司(上海);两性霉素(AMB)、Dendrimer、甲酸(色谱级)购自Sigma(美国);放射免疫沉淀法裂解液(RIPA裂解液)购自Thermo Fisher Scientific(美国); 外膜蛋白A(OmpA)兔多克隆抗体(1∶2000, CSB-PA359226ZA01EOD)购自Cusabio(美国); HRP-Anti-Rabbit二抗购自Santacruz(美国);底物(ECL)显影液购自Merck Millipore(德国);脂质标准品L-*α*-磷脂酰胆碱(PC, >98%)购自J&K Scientific(北京)。

硝酸纤维素(NC)膜(0.22 μm)购自Cytiva(瑞典);透析膜(3500 Da)购自Solarbio(北京);水性微孔过滤膜(0.45 μm)购自津腾(天津)

### 1.2 Nissle的培养和Nissle细菌培养液预处理

Nissle的培养:复苏Nissle细菌,37 ℃培养过夜后进行平板划线,培养形成单菌落。挑取单菌落于试管中进行培养,待培养基浑浊后转移到培养瓶中扩大培养至培养基再次浑浊,呈现半透明状态。为验证甘氨酸对Nissle分泌OMV的促进作用,在扩大培养步骤时,向实验组对应的培养瓶中加入培养基质量分数为1%的甘氨酸粉末,其他步骤同上。

分别收集两组Nissle细菌培养液进行以下处理:6000 g下离心10 min去除细菌和细菌碎片沉淀,得到的细菌培养上清液采用0.45 μm滤膜过滤,进一步去除细菌碎片和大的囊泡。

### 1.3 ADSP-NC膜的制备

ADSP溶液:向12 mg AMB中加入500 μL DMSO,在超声下充分溶解。依次加入240 mg EDC、144 mg NHS和50 mL MES于锥形瓶中,混合均匀后,加入上述AMB溶液和10 μL Dendrimer,避光条件下磁力搅拌过夜。裁取2个15 cm大小的透析膜,水煮活化15 min后,加入上述制备的溶液进行透析,并间隔1、2、2、4 h更换透析液(超纯水)。透析结束后,在4 ℃条件下,保存于避光离心管中。

ADSP-NC膜的制备:裁取5 cm×5 cm大小的NC膜,活化后加入ADSP溶液,于摇床上孵育4 h以上。孵育结束,加入PBS洗去ADSP-NC膜上未结合的ADSP,制备的ADSP-NC膜保存于PBS中。

### 1.4 ADSP-NC膜富集细菌OMV

采用制备的ADSP-NC膜并结合抽滤装置对细菌培养上清液中的OMV进行高通量富集,得到吸附有OMV的ADSP-NC膜。为减少ADSP-NC膜表面的非特异性吸附,向吸附有OMV的ADSP-NC膜上加入PBS洗涤10 min。富集OMV过程中,抽滤步骤所用的滤膜为制备的ADSP-NC膜,目的是通过真空泵的吸力,使细菌培养上清液快速流经ADSP-NC膜,加快OMV与膜的特异结合。相比传统的直接孵育方法,该法可在短时间内对大量细菌培养上清液中的OMV进行富集。

碱性条件下洗脱结合的OMV:将膜剪碎于EP管中,加入适量4 mmol/L氨水溶液涡旋洗脱OMV, 5000 g离心10 min,取上清液,调节pH至中性后于-80 ℃保存。

### 1.5 OMV的表征

利用BCA蛋白质定量试剂盒对甘氨酸诱导前后的OMV溶液进行蛋白质浓度测定。

#### 1.5.1 免疫印迹分析(WB)和银染

对诱导前后的OMV进行免疫印迹分析,考察细菌OMV富集情况,选用的一抗为OMV标志蛋白OmpA。按照每孔15 mg蛋白质计算所需样品体积,保证每孔蛋白质质量一致(每组样品数为3个,即*n*=3)。

#### 1.5.2 OMV颗粒数的测定

在相同的采集时间(30 s)下,采用纳米流式检测仪依次对甘氨酸诱导前后OMV样品进行颗粒数的测定。为检测样品溶液中OMV的纯度,分别向两组样品中加入样品体积10%的RIPA裂解液,并再次对颗粒数进行测定。颗粒数测定前,需根据实际情况对OMV样品进行必要稀释。

#### 1.5.3 OMV的粒径和Zeta电位分析

依次取1 mL富集的细菌OMV样品于对应测定管中,使用纳米粒度和Zeta电位分析仪进行粒径和电位检测。设定温度和稳定时间并选择对应待测材料性质后进行粒径的测定,并记录粒径分布、均值和多分散指数(PI)。Zeta电位测定步骤同上。

### 1.6 非靶向脂质组学分析

#### 1.6.1 样本预处理

在后续的非靶向脂质组学中,为探究实验组和对照组OMV在相同OMV颗粒数下的差异脂质,采用纳米流式仪进行计数后,分别取对应体积的OMV样本进行真空冷冻干燥。采用甲醇-MTBE-水溶剂体系提取细菌OMV中的脂质,步骤如下:向OMV冻干样品中加入100 μL甲醇溶液,液氮下反复冻融3次使OMV破碎裂解;加入250 μL MTBE涡旋振荡15 min,加入100 μL去离子水分层,上层脂质10000 g离心10 min后,取上清液冷冻干燥于-20 ℃保存。上样分析前进行样品复溶,复溶溶剂为A(氯仿-甲醇,2∶1)和B(ACN-IPA-水,65∶30∶5),以1∶2的体积比混合,总体积为180 μL,离心后取上清液于进样小瓶中。

#### 1.6.2 色谱条件

色谱柱:ACQUITY UPLC BEH C18色谱柱(50 mm×2.1 mm, 1.7 μm, Waters),柱温为50 ℃;流速0.3 mL/min;进样量为5 μL;流动相:(A)ACN-水(3∶2)和(B)IPA-ACN(1∶1);梯度洗脱程序:0~2.0 min, 40%B~43%B; 2.0~2.1 min, 43%B~50%B; 2.1~8.0 min, 50%B~54%B; 8.0~8.1 min, 54%B~70%B; 8.1~10.0 min, 70%B~99%B; 10.0~10.1 min, 99%B~40%B; 10.1~12.0 min, 40%B。

#### 1.6.3 质谱条件

采用电喷雾离子源(ESI),正离子扫描模式检测。离子源温度:300 ℃;锥孔电压为40 V,二级碰撞能量(CE)范围为20~50 V,毛细管电压1000 V;干燥气(氮气)、碰撞气(氩气)的流速分别是50 L/h和800 L/h,雾化气压强为650 kPa,淌度气体为氦气。采用MS^E^模式进行数据采集。采用的脂质标准品为PC和SM。

### 1.7 数据处理与统计分析

利用MS-DIAL脂质分析软件(Mass Spectrometry Data Independent Analysis for Lipidomics,百泰派克生物科技有限公司,北京)对实验组和对照组数据进行定性,获得精确质荷比(*m/z*)、保留时间(RT)、脂质类型等信息,然后通过LIPID MAPS数据库(LIPID Metabolites and Pathways Strategy, National Institutes of Health,美国)对脂质的结构信息以及分类进行核对。

## 2 结果与讨论

本研究总体流程如图1所示,首先通过ADSP-NC膜对Nissle细菌培养液进行OMV的高通量捕获,为验证富集效率,依次对富集的OMV进行表征;为探究在细菌生长过程添加甘氨酸对细菌分泌的OMV脂质成分的影响,利用UPLC-IMS-QTOF-MS对提取的OMV脂质成分进行分离分析,进行非靶向脂质组学分析。

**图1:**
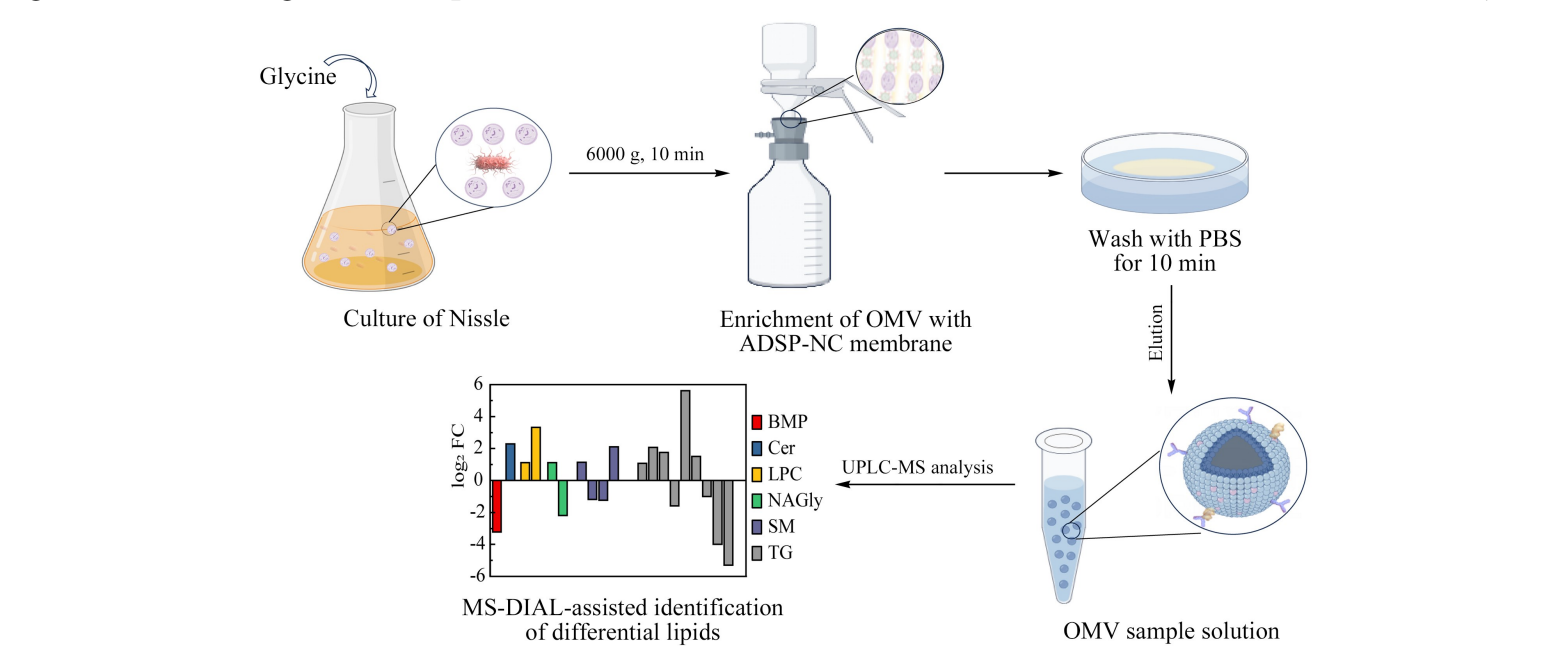
细菌OMV的富集和脂质组学分析流程

### 2.1 OMV的WB、银染、颗粒数、纯度和粒径分布分析

为评估OMV的富集情况,对甘氨酸诱导前后的OMV进行蛋白质浓度检测,结果分别为0.3 mg/mL和0.6 mg/mL。OmpA是Nissle细菌OMV的表面特异蛋白,维持细菌外膜的完整,参与OMV的形成和分泌过程。为验证OMV是否成功富集,对其进行WB分析,由图2a可知,甘氨酸诱导组和非诱导组均可见OmpA双蛋白质条带,这标示着OMV的成功富集。甘氨酸诱导组中OmpA蛋白质条带颜色清晰可见,而非诱导组的蛋白质条带颜色较浅,通过Image J软件进行灰度分析,得到两者对应的蛋白质条带的灰度值及比值。结果表明,在细菌培养过程中加入甘氨酸可促进OMV分泌。WB和银染都是检测蛋白质条带的常用手段,由图2b可知,在保证每孔蛋白质质量一致时,诱导前后的OMV银染条带在35 kDa附近处均清晰可见,两组间及组内平行样本的OMV条带均无明显差异。结合WB来看,表明在相同蛋白量条件下,未经甘氨酸诱导的细菌OMV中杂蛋白较多。

**图2:**
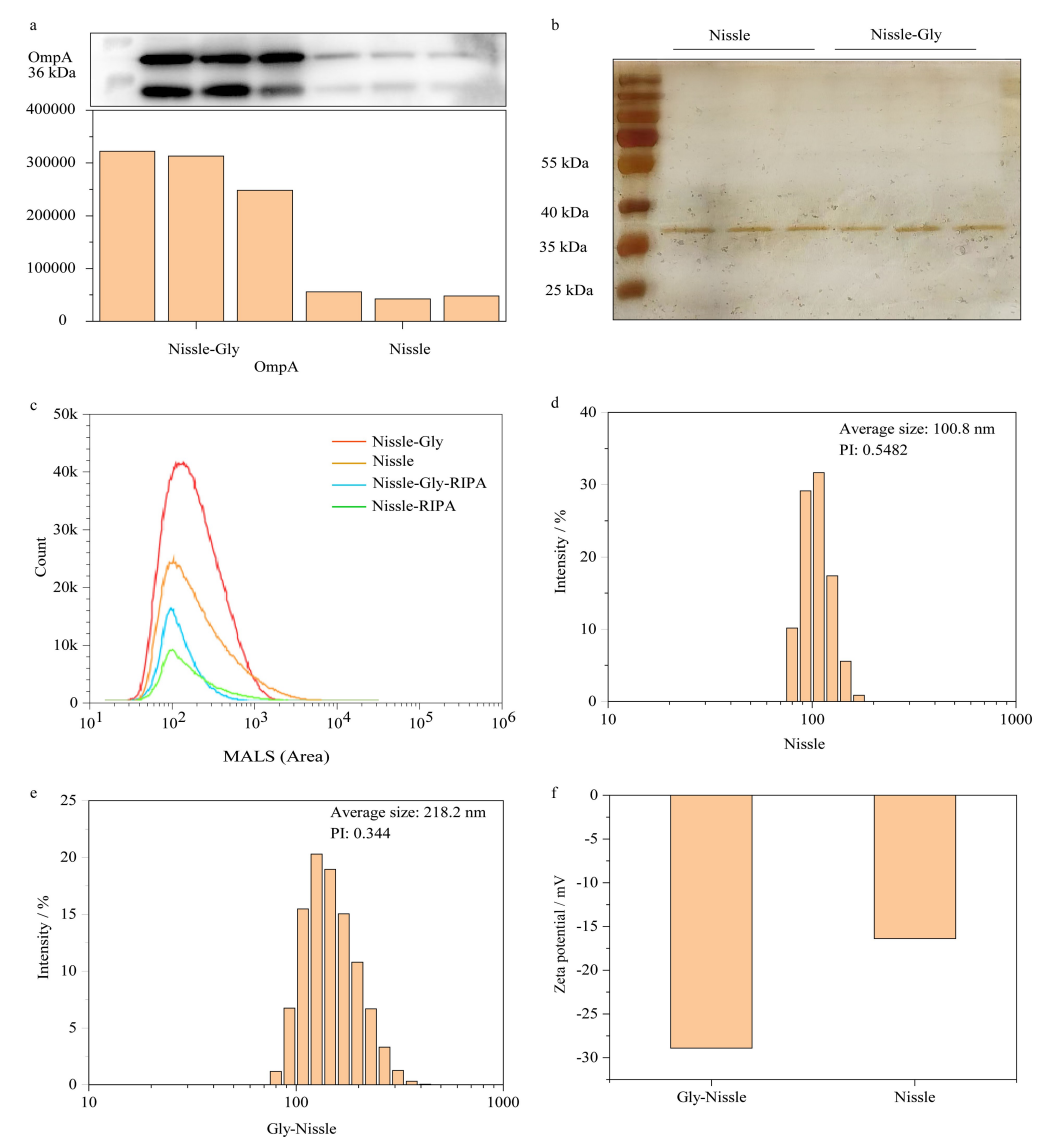
甘氨酸诱导前后的OMV表征

采用纳米流式检测仪对OMV的颗粒数和纯度进行测定,由预实验可知,甘氨酸诱导组细菌OMV数约为非诱导细菌OMV数量的10倍,为考察相同数量级下制备的OMV样品溶液的OMV纯度,诱导组测试前进行10倍稀释。RIPA裂解液可破坏囊泡结构,对加入RIPA裂解液前后的颗粒数进行测定可大致得出OMV样品中OMV的纯度。由图2c可知,稀释10倍的甘氨酸诱导组进行RIPA裂解前颗粒数为2.76×10^9^个/mL,裂解后的颗粒数为5.67×10^8^个/mL,可得诱导组OMV样品纯度为79.5%;非诱导组裂解前的颗粒数为1.56×10^9^个/mL,裂解后的颗粒数为4.14×10^8^个/mL,非诱导组OMV样品纯度为73.5%。

结果显示,当两组样品OMV颗粒数在同一数量级时,甘氨酸诱导组OMV的纯度稍高于非诱导组,同时两组OMV样品中OMV纯度均较高,表明囊泡结构完整性良好。

对细菌OMV的粒径大小和Zeta电位进行测定,结果如图2d和2e所示,非诱导组的粒径尺寸为80~150 nm,PI为0.5482;甘氨酸诱导组OMV整体粒径尺寸增大,粒径为90~200 nm,分布较为均匀,PI为0.344;表明在细菌培养过程中添加甘氨酸对分泌的OMV大小和稳定性具有一定影响,分泌的OMV不易聚集,分散性良好。图2f是测定的Zeta电位结果,甘氨酸诱导组的数值为-28 mV,非诱导组的数值为-17 mV, OMV的双层磷脂膜结构导致其表面带负电。Zeta电位是表征胶体分散系稳定性的重要指标,Zeta电位的绝对值越高,体系越稳定,表明甘氨酸诱导后所分泌的细菌OMV的稳定性增强。

### 2.2 UPLC-IMS-QTOF-MS分析

将制备的脂质冻干粉复溶,离心后取上清液于进样小瓶中,复溶剂作为空白对照。离子淌度技术可辅助传统的LC-MS对检测到的分析物进行多维表征,在代谢组学、蛋白质组学分析中均有应用。选取淌度时间(drift time) 20~180 Bins, *m/z* 500~1000,分别调取诱导前(图3-Nissle)和诱导后(图3-Nissle-Gly)对应的离子淌度图,对其中的差异脂质进行标记。

**图3:**
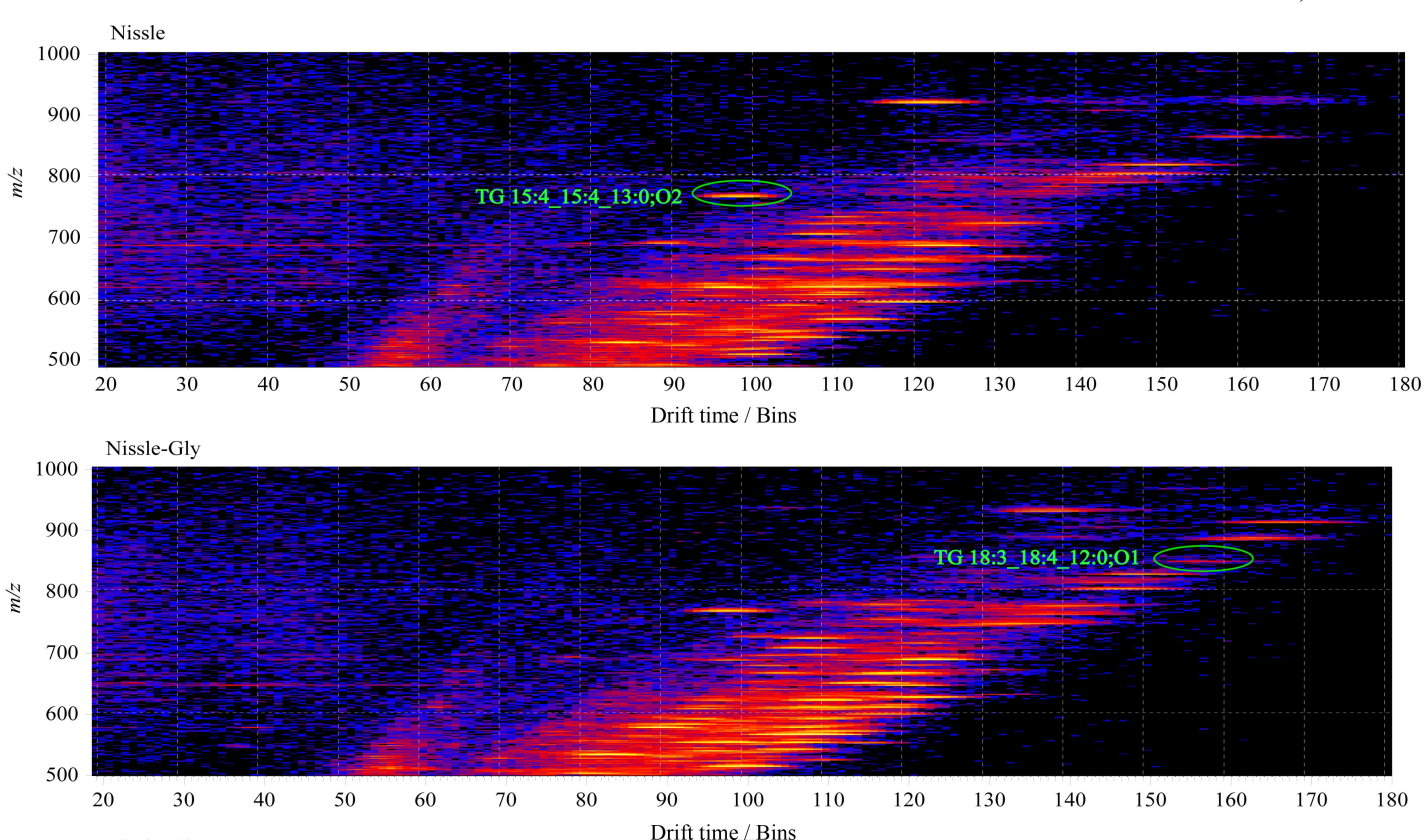
甘氨酸诱导前后的细菌OMV进行离子淌度质谱分析

### 2.3 甘氨酸诱导前后OMV脂质差异表达分析

研究发现,向Nissle培养基中补充甘氨酸,与非诱导组OMV相比,不仅可显著增加分泌的OMV数量,诱导组OMV的内膜蛋白RodZ、胞浆蛋白Crp和AdhE的表达水平也显著增加。我们通过多变量分析进一步研究总体水平上的脂质紊乱,结果发现,甘氨酸诱导后脂质的表达也发生了显著变化。

为探究在Nissle培养过程中添加甘氨酸对OMV脂质组成分的影响,利用非靶向脂质组学技术分析了甘氨酸诱导前和诱导后的OMV脂质组,共鉴定出820个脂质分子,主要有甘油三酯(TG)、甘油二酯(DG)、神经酰胺(Cer)、SM、*N*-酰基乙醇胺(NAE)、PC、双(单酰基甘油)磷酸酯(BMP)、*N*-酰基牛磺酸(NATAU)、溶血磷脂酰胆碱(LPC)等。

采用MS-DIAL脂质数据分析软件,通过*m/z*、RT、MS/MS、峰面积一一匹配,实现OMV样品中脂质成分的定性定量。为考察软件和仪器测定的准确性,采用PC和SM脂质标准品进行验证,结果表明测定脂质相对分子质量与精准相对分子质量之间误差为8×10^-6^(8 ppm)。结合LIPID-MAPS数据库进行脂质分类,包括以下8类,分别是脂肪酸类(fatty acids, FA)、甘油脂类(glycerolipids, GL)、醇脂类(sterol lipids, ST)、鞘脂类(sphingolipids, SP)、甘油磷脂类(glycerophospholipids, GP)、糖脂类(saccharolipids, SL)、孕烯醇酮脂(prenol lipids, PR)和多聚乙烯类(polyketides, PK)。由图4a可知,本次检测到的820个脂质成分中,按照含量高低排序,依次是GL、FA、SP、GP、SL、ST,其中GL类脂质组分有462个,所占比例为56.4%。由图4b可知,一共鉴定出24种脂质,数量最多的6种脂质分别是TG、DG、Cer、SM、NATAU、NAE,比例为28.4%、26.9%、6.8%、6.5%、5.2%、5.0%,分别归属于GL、GL、SP、ST、FA、FA大类。

**图4:**
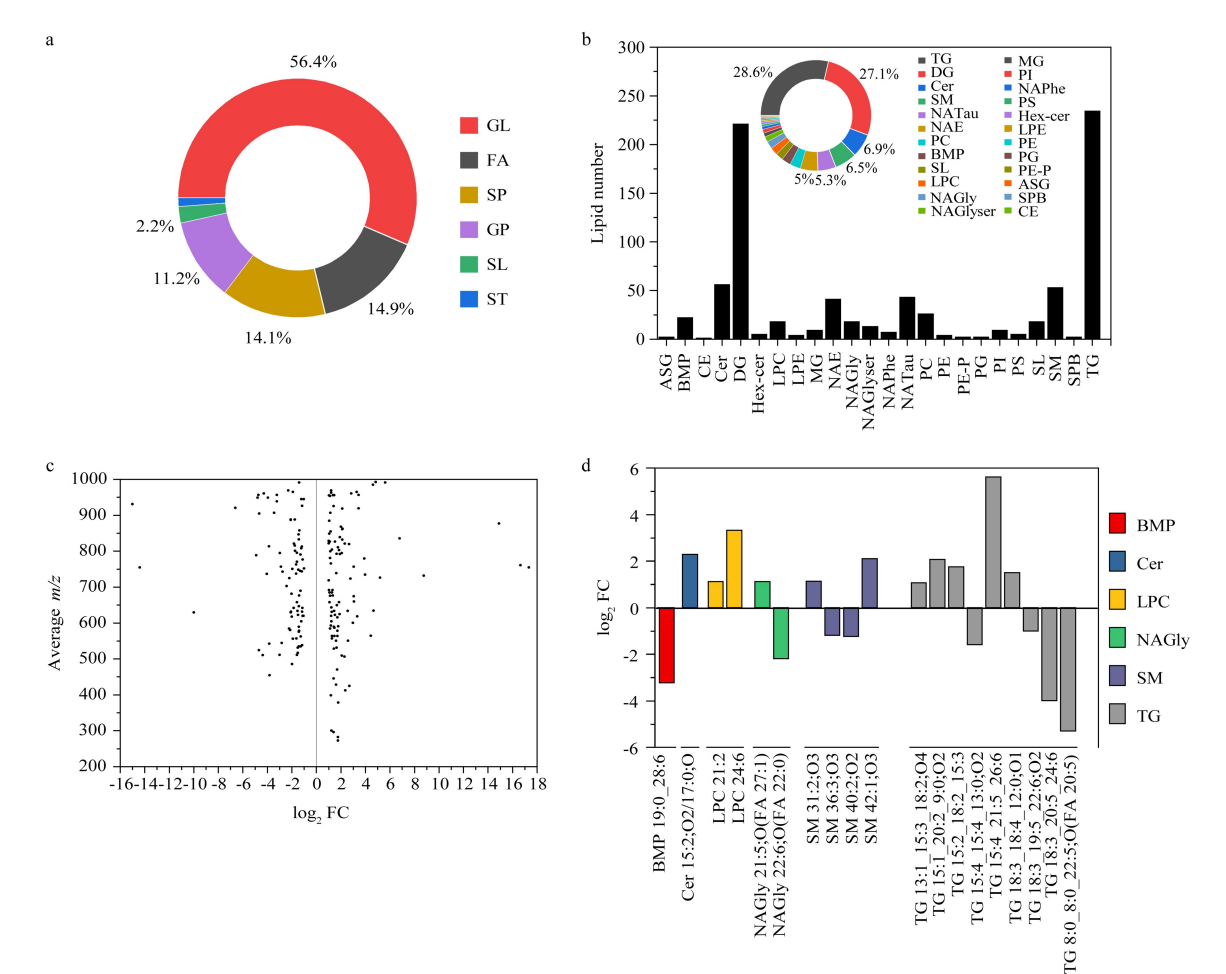
诱导前后的细菌OMV的脂质组学分析

对定性到的脂质成分进行差异脂质的筛选。依次计算诱导前后两组脂质成分对应峰面积的比值(fold change)和*P*-value,在满足*P*-value<0.05条件下筛选FC>2和FC<0.5,得到甘氨酸诱导后脂质代谢表达发生改变的脂质,按照质荷比大小绘制差异脂质散点分布图(图4c)。FC>2和FC<0.5分别表示为该物质在甘氨酸诱导后表达上调和该物质在甘氨酸诱导后表达下调。图4c中右侧为表达上调的脂质成分在甘氨酸诱导组中高度表达,反之,甘氨酸诱导后部分脂质表达量明显下降。分析此图发现整体差异脂质的log_2_ FC的大小范围为1~4,即差异脂质表达上下调的差异倍数为2~16。从得到脂质离子的*m/z*来看,多数表达差异脂质组分的*m/z*分布在500~1000区间,只有脂质表达上调中发现部分*m/z*为300~500的脂质离子。

考虑到实验的严谨性,再次对MS-DIAL脂质数据库匹配的差异脂质结果进行筛选,只保留匹配分数较高的脂质组分。甘氨酸诱导前后具有差异的脂质成分有19个,上下调脂质数量分别为11和8个。因此,在上调差异脂质组分中,共发现5种脂质组分,分别为TG、SM、Cer、LPC、NAGly。在下调差异脂质组分中发现4种脂质,分别为TG、BMP、SM、NAGly。此外还发现了一些特殊脂质成分,它们的表达具有一定规律性,在甘氨酸诱导后表达只发生上调的脂质有LPC和Cer,如LPC 24∶6、LPC 21∶2、Cer 15∶2;O2/17∶0;O;表达只出现下调的脂质有BMP,如BMP 19∶0_28∶6,图4d为甘氨酸诱导后脂质表达量发生改变的脂质成分。

## 3 结论

本研究利用ADSP-NC膜对甘氨酸诱导前后的细菌培养上清液中的OMV进行富集,并对富集的OMV进行了一系列表征,包括WB、银染、蛋白质浓度、粒径分布和Zeta电位以及细菌OMV颗粒数和纯度,结果表明细菌OMV成功富集。进一步对其富集的OMV进行脂质提取,基于UPLC-IMS-QTOF-MS和MS-DIAL软件对其进行非靶向脂质组学分析。结果表明两组的差异脂质组分的种类大致相同,但不同类型的脂质变化较大,后期可结合其他研究(如蛋白质组学)进行综合分析,对其差异脂质成分进行通路和机制的分析,探究其作为新型药物递送载体应用的科学依据。
